# Insularity and Aridity as Drivers of Mandibular Disparity in *Thylamys elegans* (Waterhouse, 1839) from Populations of the Atacama Desert, Chile

**DOI:** 10.3390/ani12091179

**Published:** 2022-05-04

**Authors:** José I. Arriagada, Hugo A. Benítez, Frederick Toro, Manuel J. Suazo, Paulette Abarca, Jhoann Canto, Yerko A. Vilina, Franco Cruz-Jofré

**Affiliations:** 1 Escuela de Medicina Veterinaria, Facultad de Recursos Naturales y Medicina Veterinaria, Universidad Santo Tomás, Chile, Limonares 190, Viña del Mar 2561780, Chile; joseignacioarriagadadiaz@gmail.com (J.I.A.); frederick.toro.c@gmail.com (F.T.); 2 Laboratorio de Ecología y Morfometría Evolutiva, Centro de Investigación de Estudios Avanzados del Maule, Universidad Católica del Maule, Talca 3466706, Chile; hbenitez@ucm.cl; 3 Centro de Investigación en Recursos Naturales y Sustentabilidad (CIRENYS), Universidad Bernardo O’Higgins, Avenida Viel 1497, Santiago 8370993, Chile; 4 ONG Panthalassa, Red de Estudios de Vertebrados Marinos en Chile, Santiago 8370003, Chile; 5 Instituto de Alta Investigación, Universidad de Tarapacá, Casilla 7D, Arica 1000000, Chile; suazo.mj@gmail.com; 6 Programa de Magíster en Ciencias, Mención Biodiversidad y Conservación, Universidad de Valparaíso, Gran Bretaña 1111 Playa Ancha, Valparaíso 2340000, Chile; paulette.abarca.m@gmail.com; 7 Centro de Rescate y Rehabilitación Fundación ÑAMKU, Concón 2510000, Chile; 8 Área de Zoología de Vertebrados, Museo Nacional de Historia Natural, Santiago 8500000, Chile; jhoann.canto@mnhn.cl; 9 Whalesound Ltd., Punta Arenas 6200000, Chile; yerkovil@yahoo.com; 10 Laboratorio de Genética y Evolución, Departamento de Ciencias Ecológicas, Facultad de Ciencias, Universidad de Chile, Santiago 8330015, Chile

**Keywords:** insular populations, geographical isolation, environmental gradient, geometric morphometrics, prey species richness, mandibular morphology

## Abstract

**Simple Summary:**

The following article studied the environment influence between island and mainland in an endemic marsupial from Chile the mouse-opossum Thylamys elegans (Waterhouse, 1839). Its important to understand that the isolated habitat could affect the pattern of morphological evolution in organism due to millions of years of being separated from the mainland. In this re-search we used two methodologies to study those effects in the mandible of this marsupial, first a biomechanical methodology which was used to correlate it with the diet, and a second called ge-ometric morphometrics which combine the geometry and biology to identify the principal changes in the morphology. Our results showed that Island populations have more disparity in the mor-phology in comparison to the mailand differences that could be related to the arid environment and other characteristic of the island. Concluding that one of the possible reason of the evolutionary history of this Chilean mouse-opossum was processes of natural selection by a process of coloniza-tion of island after vicariance denominated founder effect.

**Abstract:**

Island ecosystems differ in several elements from mainland ecosystems and may induce variations related to natural selection and patterns of adaptation in most aspects of the biology of an organism. *Thylamys elegans* (Waterhouse, 1839) is a marsupial endemic to Chile, distributed from Loa River to Concepción. Historically, three subspecies have been described: *Thylamys elegans elegans*, *Thylamys elegans coquimbensis* and *Thylamys elegans soricinus*. For this research, two morphometric approaches and a biomechanical model were used to compare the mandible shapes and biomechanics between two Chilean mouse opossum populations belonging to different subspecies: one from the coastal desert of Chile (*T. e. coquimbensis*) and the other from the central inland region (*T. e. elegans*). Additionally, mandibles of insular populations found in the Reserva Nacional Pinguino de Humboldt (RNPH)), from which the subspecies association is unknown, were also included. The results showed that insular populations have differences in mandibular shapes, sizes and biomechanical characteristics compared to continental populations, which may be related to environmental variables like aridity and vegetation cover, prey type, insularity effects and/or the founder effect on micromammals, apart from vicariance hypotheses and other selective pressures.

## 1. Introduction

Islands are relevant locations for speciation and adaptive radiation studies [1,2,3]. These scenarios are where micromammals become outstanding models due to their low capacity to go across barriers (e.g., marine barriers) and/or long geographical distances, which is related to phylogeographic concepts such as isolation and the founder effect [4,5]. Islands are natural laboratories in which biogeographic hypotheses can be tested [6] in order to establish general rules for body shapes and sizes. On example of this is the “island rule”, which postulates a tendency toward gigantism in insular micromammal populations due to the absence of predators and a more intense intraspecific competition [7]. However, some characteristics like predator presence/absence and resource availability may change some of these patterns [8]. Thus, the intensity of the morphological changes between islands may depend on the size of the island and how distant it is from the continent [9]. The island rule was recently tested in rodents of the Fuegian archipelago in the south of Chile, where the observation of an incongruent pattern between co-distributed species was attributed to differences in their dispersal capacity [10]. The coastal islands in the south end of South America have a peculiar history, since they are covered by ice and/or they modified their connections with the continent during the last glacial cycle (35 Kyr BP) [11,12]. In other areas uncovered by ice, the continental shore was affected by these global changes, increasing between 100 and 150 m in height from sea level in the last 20 Ka [13], moving the coastal line 7 km inland [14] and therefore causing some islands that were formed near the continent. The island effect can be directly related to the morphology of organisms, as island populations present a wide range of shapes, sizes and biomechanical traits different from those observed in continental populations. This has been observed in rodents, shrews and insects [15,16,17,18,19,20].

The order Didelphimorphia is the most diverse group of marsupials that currently inhabit the American continent (116 species; 18 genera) [21,22]. It has a single family, Didelphidae, characterized by diverse morphotypes and ecological niches in its distribution range, covering from Southern Canada to the Patagonia [23], which entails a wide variety of habitats and environmental conditions. Only two didelphid species can be found in Chile: *Thylamys pallidior* (fat-tailed or northern mouse opossum) and *Thylamys elegans* (Chilean mouse opossum), the latter being endemic to Chile. Their distribution is from the Loa River to the Concepción region [24], covering an aridity gradient that includes the Atacama Desert in the north, scrublands and sclerophyll forest in the central zone and temperate forests in the south. *T. elegans* has arboreal habits and a mainly insectivorous diet [25], although it has also been described as opportunistic (i.e., able to exploit different resources in the environment based on availability) [25,26,27]. Historically, three subspecies have been described: *T. elegans elegans* (Valparaíso locality), *T. elegans coquimbensis* (Paiguano locality, Center-North Chile) and *T. elegans soricinus* (Valdivia) [24,27,28]. However, the genetic correspondence of these taxonomical units is unclear, since a larger number of lineages have been described in this geographical distribution, determining that some rivers act as geographic barriers for the genetic flow between populations and/or subspecies [29,30]. In the north of Chile, in the islands of the Humboldt Penguin National Reserve (29° S), *T. elegans* populations have been described [31,32], but they have not been previously studied or assigned to any of the subspecies mentioned above. These insular populations are within the plant formation of the Huasco Coastal Desert [33], where *T. e. coquimbensis* is distributed. This geographic zone is characterized by the presence of small bushy vegetation, mainly cactaceae of the genera *Copiapoa*, *Eulychnia* and *Neoporteria* and shrubs of the genera *Nolana*, *Oxalis* and *Heliotropium* [34,35]. Further south, in the central zone of Chile, *T. e. elegans* is distributed in an area with a Mediterranean climate and sclerophyll forests, with higher vegetation cover and medium-sized trees with small leaves [36]. These environments, characterized by heterogeneous vegetation, have, in turn, a contrasting biodiversity of arthropods, which are potential prey for *T. elegans*. Since mandible morphology is directly related to diet in placental and marsupial mammals [37,38], it is considered an interesting structure for adaptation and populational variability studies [39,40,41].

In general terms, the mandible shape of *Thylamys elegans* has a mandibular ramus (coronoid process) with a posterior inclination, forming an open angle with an elongated, thin mandibular body and a long molar series. These characteristics would give an advantage to crush and cut prey, and they are consistent with the aspects proposed for mammals with an insectivorous diet [37,42,43,44], for fossil insectivorous metatherian [38] and *Metachirus nudicaudatus* (Geoffroy 1803), a neotropical marsupial with an insectivorous diet [45].

In fossil marsupials, a general pattern has been identified regarding the mandible shape, where carnivorous species have a more perpendicular ramus and stronger mandibular corpus, whereas frugivorous and insectivorous species have wider mandibular angles (between the corpus and coronoid process) [37,38]. Furthermore, these diet-related patterns may be concurrent with others relative to habitat use, such as prey availability and type or arboreality, with the latter explained by the position of the head in regards to the vertical position of the body during this activity, among other factors [45,46]. In the context outlined above, the purpose of this study is to compare the morphological characteristics of *Thylamys elegans* mandibles using both geometric and linear morphometrics coupled with a simple biomechanical model in order to assess the changes in shape, size and functionality of the mandibles in two subspecies. This will allow us to evaluate the potential effect of insularity and aridity on the mandible shape and size in *T. elegans* populations inhabiting the center-north of Chile.

## 2. Materials and Methods

The material analyzed was composed of mandibles collected in the field. They were recovered from pellets found in predator perches (e.g., *Athene cunicularia*) or belonged to dead animals (including both medical or natural reasons) that arrived at the HCV-UST Wild Fauna Rescue Center (Viña del Mar, Chile) and at Ñamku Foundation. Additionally, the sample was complemented with mandibles from the Vertebrate collection of the National Museum of Natural History (MNHN Santiago, Chile). In total, 95 mandibles were analyzed: 47 right-side (RM) and 48 left-side (LM) (Supplementary Table S1 for the compositions of the mandibles per analysis), including only adult specimens in the analyses (pm3 and M4 fully erupted).

### 2.1. Thylamys elegans Subspecies

**Figure 1:**
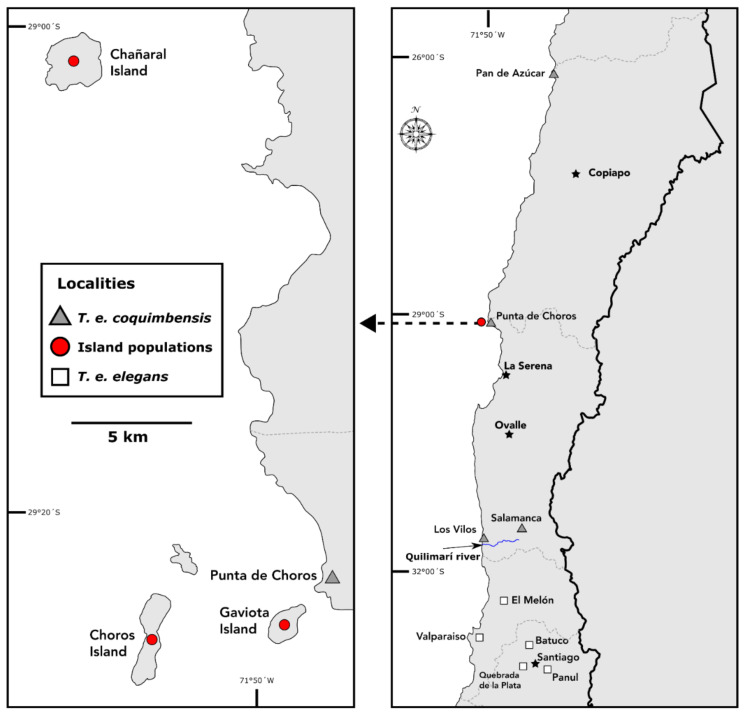
Distribution map of the samples used in this study.

Two of the three *Thylamys elegans* subspecies found in Chile were included in the analyses: *Thylamys elegans elegans,* distributed in the center of Chile between the Aconcagua and Maipo Rivers, and *Thylamys elegans coquimbensis*, with a distribution north of the Quilimarí River covering the coastal desert in the Atacama zone (Quebrada El León and Llanos de Challe) and Coquimbo region [24,29]. Specimens by subspecies and location are detailed in the Supplementary Materials (Supplementary Table S1). The analyses included 27 mandibles from three islands (Choros, Gaviota and Chañaral) located between the Coquimbo and Atacama regions. Individuals in these localities have not been assigned to any of the before-mentioned subspecies, but their distribution is closer to *T. e. coquimbensis*. The distances from the islands to the continent are less than 10 km (Choros island 6 km, Gaviota island 0.4 km and Chañaral island 8 km), and all of them are under 510 ha in size (Figure 1). There was no information on the sex of each specimen, so its effect was not evaluated. However, it has been reported that there are no cranial or mandibular differences between males and females in *T. elegans* [47].

### 2.2. Linear Morphometrics

**Figure 2:**
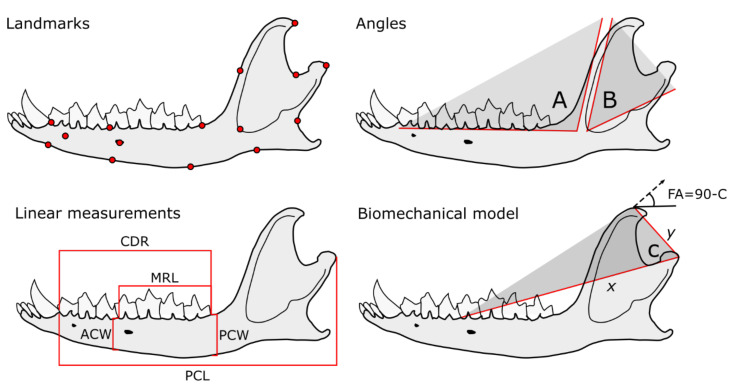
Summary of landmarks, angles and measures of *Thylamys elegans*, the descriptions of angles A and B are defined in the Section 2.2 and the angle C in Section 2.4.

Linear mandibular measurements were taken, based on previous works on Didelphidae [39,48,49]. The mandible was characterized with the following measures: partial corpus length (PCL), length of complete dental row (CDR), length of mandibular molar row (MRL) and two measurements of the mandibular width (below the third premolar and below the fourth molar). The anterior corpus width (ACW) is the distance from the alveolus of the third premolar to its projection in the ventral margin of the mandibular body, while the posterior corpus width (PCW) is the distance between the fourth molar alveolus and the ventral mandibular margin (Figure 2).

In addition, two angles were measured: angle A, defined by the anterior border of the mandibular ramus and the dental alveolus line (mandibular angle), and angle B, which is formed within the masseteric fossa and outlined by the dorsal and ventral borders of this fossa (Figure 2). All pictures were taken with a digital camera (CANON Powershot SX60), and we included a rule to scale the pictures. All the measurements were performed using the software program TPSdig2 v.2.31 [50].

### 2.3. Geometric Morphometrics

**Figure 3:**
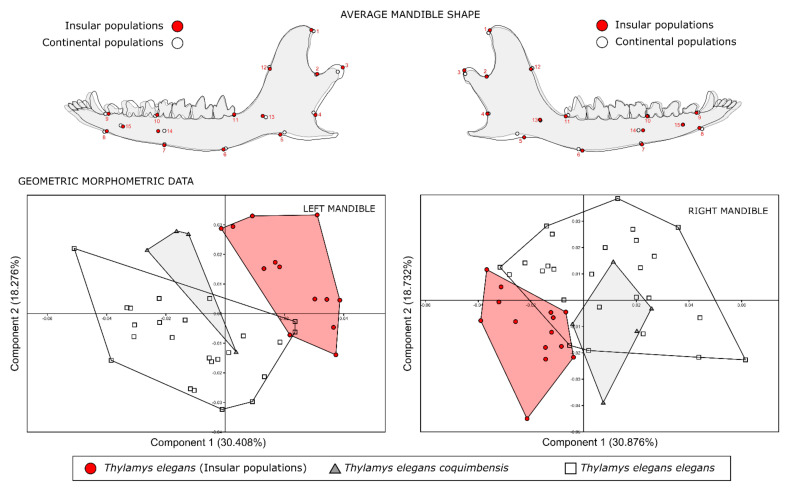
(**Left**) and (**Right**) Average mandible shapes of insular and continental populations of *Thylamys elegans* and their corresponding Principal Component Analysis. Legend: red circles, insular populations of *Thylamys elegans*; grey triangles, *Thylamys elegans coquimbensis*; white squares, *Thylamys elegans elegans*.

An image in the lateral view was taken from each mandible. The shape of the structure was characterized with 15 landmarks (Figure 2 and Figure 3) digitized with the software program TPSdig2 v.2.31 [50]. The landmark definitions are as follows: (1) tip of coronoid process, (2) most concave point between the coronoid and articular processes, (3) caudal-most point of the articular process, (4) most concave point between the articular and angular processes, (5) intersection between the ventral borders of the articular process and mandibular corpus, (6) and (7) mandibular body ventral curvature (positioned at the same distance between LMK 5 and 8), (8) perpendicular projection of landmark 9 onto the mandibular border, (9) first premolar (PM1) rostral end, (10) rostral end of the first molar (M1), (11) posterior end of the fourth molar (M4), (12) midpoint between landmarks 1 and 11 on the anterior border of the coronoid process, (13) tip of the masseteric fossa angle and (14) and (15) mental foramina. A Procrustes superposition analysis was conducted using MorphoJ v1.06d [48]) in order to remove the scale, rotation and translation information from the mandible shape, thus creating our shaped variables [51]. The Procrustes coordinates and the centroid size of the specimens were calculated using MorphoJ v1.06d software and were used as shape variables in the statistical analyses. To evaluate the correct classification between the groups, a discriminant analysis and its classification accuracy were assessed using cross-check validation procedures performed in MorphoJ v1.06d [48].

### 2.4. Biomechanical Model

To relate mandible shape with bite force, the simple biomechanical model proposed by Young et al. [52] was used, following the modifications by Cornette, Herrel, Cosson, Poitevin and Baylac [15]. This model calculates the biomechanical potential and estimates the bite force by simplifying the mandible as a simple lever. Three different measures were used for this: *x*, distance from the articular condyle to the point located between the first and second molar; *y*, distance from the articular condyle to the most dorsal point of the coronoid process, and angle C that was delimited by distances *x* and *y*. The force angle (FA) is calculated as FA = 90 − C. The mechanical potential (MP) is calculated as MP = *y*/*x* cosine (FA) (Figure 2) and represents the force directed at a right angle by the temporalis muscle on the coronoid process.

### 2.5. Comparisons between Groups

Individuals were classified into three groups: one of them with insular populations of this species and the other two including the subspecies *T. e. coquimbensis* and *T. e*. *elegans*. In order to summarize the shape variations between the studied populations, we performed a Principal Component Analysis (PCA) on the shape variables [53,54] in the program MorphoJ v1.06d [55]. Subsequently, to quantify the effect of size on the mandible shape, a multivariate regression was conducted using the centroid size as the independent variable and the mandible shape as the dependent variable (regression score 1). Then, a size-corrected PCA was carried out on the residuals of this multivariate regression, which contained shape information free from allometric size [56,57].

The program PAST4 [58] was used for analyses on the linear morphometrics dataset to identify the subset of measurements whose range of values better described the different groups. With the linear measures of the mandible, a discriminant analysis was performed, and the group assignment was cross-validated by the leave-one-out cross-validation (jackknifing) procedure. After that, a PERMANOVA test was conducted using 10,000 permutations to compare the three groups. A statistical *t*-test (10,000 permutations) was performed to make comparisons between the means of the linear measurements, angles (A and B), mechanical potential and centroid size in each one of the groups. Finally, the angle dimensions were related by means of linear regression (angle A-dependent variable and angle B-independent variable).

### 2.6. Characterization of Potential Prey: Arthropods, Fruits and Seeds

Since arthropods comprise 90% of the mouse opossum diet [25], arthropod richness was compared between coastal desert areas (subspecies *coquimbensis* and insular populations) in Chile and areas with a Mediterranean environment (subspecies *elegans*). Body size information was collected from the literature of Chilean arthropods, and prey species were classified into 5 size categories (in millimeters): very small (1–7), small (8–15), medium (16–25), big (26–40) and very big (41–100). Fruits and seeds, which account for the remaining 10% of the mouse opossum diet composition, were classified into two categories (fleshy; not fleshy) according to Yu et al. [59] (Table 1).

### 2.7. Relation between Environment and Mandible Shape and Size

A Pearson correlation coefficient was calculated in PAST4 [58] to evaluate whether there is a relationship between mandible shape/size and environmental variables. The variables were used by pairs; first: average centroid size, PC1 eigenvalue and PC2 eigenvalue per locality and second: environmental indexes of vegetation (NDVI), mean annual temperature in °C × 10 (bio1) and annual rainfall in mm (bio12) from the localities where samples were found (the latter two aspects were taken from WorldClim 1.4 (historical climate conditions)) [60].

## 3. Results

The first two principal components (PC1 and PC2) accounted for 48.6% of LM- and 49.6% of RM-shaped variations, and differences between the insular and continental populations were observed along with PC1. Furthermore, there is a superimposition of mandible shapes of subspecies *T. e. elegans* and *T. e. coquimbensis* between the first two dimensions of the morphospace (Figure 3). The differences have shown that both the tip of the coronoid and the articular condyle are positioned more anteriorly in the insular individual, associated with a larger amplitude in the masseteric fossa, a closer angle between the coronoid process and the mandibular body (Angle A). A displacement to the posterior of LMK 11 is related to the posterior displacement of the fourth molar. Finally, a posterior movement of LMK 8 and 9 is related to the shortening of the mandible. 

**Figure 4:**
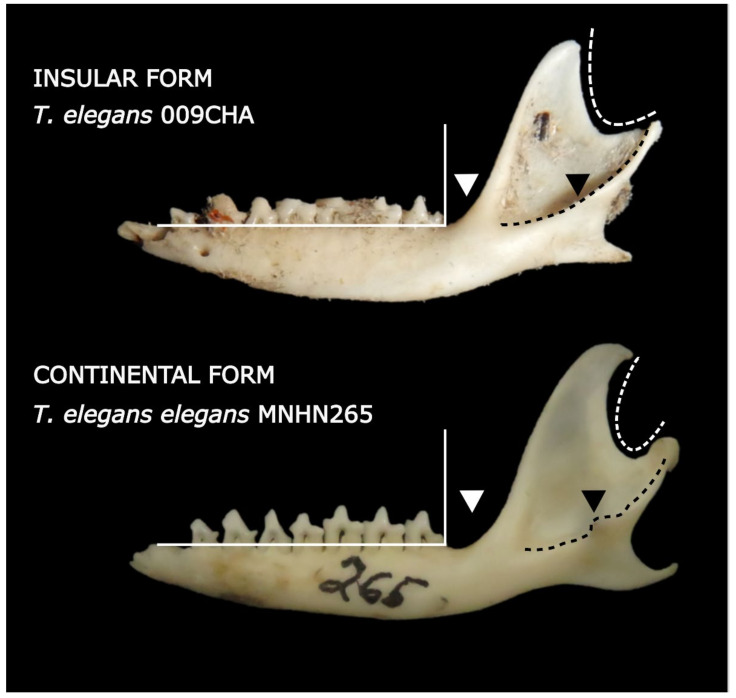
Comparison of representative mandibles of insular (**top**) and continental (**bottom**) populations of *Thylamys elegans*. Morphological features: white triangle: retromolar fossa; black triangle: masseteric tuberosity. Continuous white lines are perpendicular to the molar line, and black segmented lines show the ventral border of the masseteric fossa, while the segmented white lines showcase the curvature between the coronoid process and the angular process.

From a qualitative view, the morphological characters found that. in the mandibles from the islands, the retromolar fossa was smaller, with a deeper masseteric fossa, and the curvature between the coronoid process and the angular process was more open. In addition, the absence of the masseteric tuberosity was observed in most of the insular individuals analyzed in comparison to the continental mandible (Figure 4).

The allometric effect accounted for 14.07% LM and 14.08% RM of the shape variation explained by size. Additionally, the permutation test showed that the allometric effect was significant (*p* < 0.001, LM and RM), with insular individuals having smaller sizes than the continent (Supplementary Figure S1). The PCA from the residual component of the shape variation in the groups showed groupings by population, distinguishing insular individuals from continental populations (*T. e. coquimbensis* and *T. e. elegans*) (PC1) in the morphospace for both mandibles (LM and RM) (Supplementary Figure S2).

**Figure 5:**
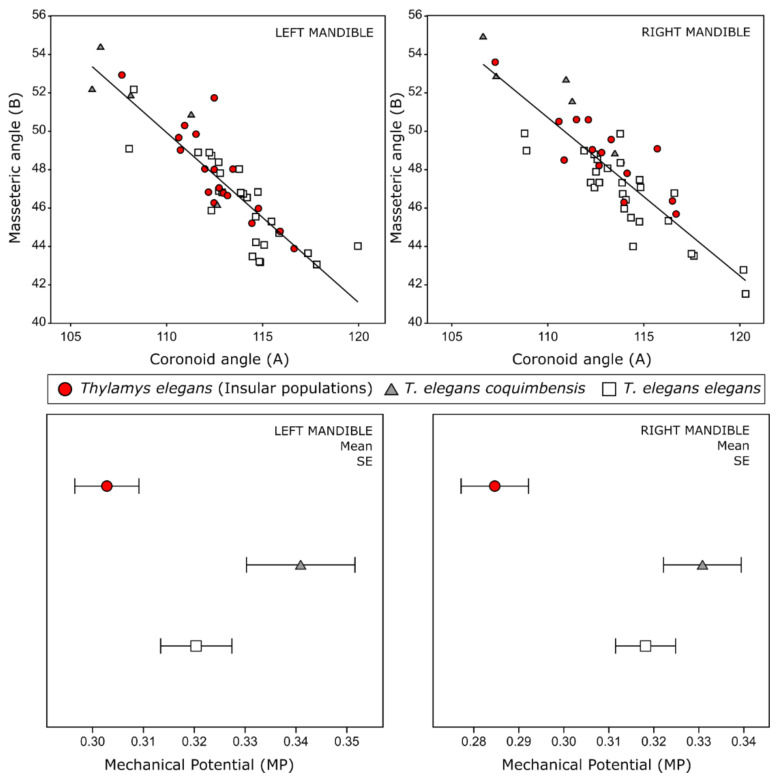
(**Top** row) Linear regressions between Angle A and Angle B for each hemimandible. (**Bottom** row) Mechanical potential mean values and standard error in the three groups analyzed. See the symbol legend in Figure 3.

The linear regression showed an inverse relationship between angle A (coronoid) and angle B (masseteric fossa) (LM: R = −0.87, *p* = 0.0001; RM: R = −0.87, *p* = 0.0001; Figure 5).

In the biomechanical model, the lowest mechanical potential was observed at the insular populations (0.28 LM–0.30 RM), whereas the highest MP was achieved by the subspecies *T. e. coquimbensis* (0.34 LM–0.33 RM) (Figure 5). This was confirmed by the comparison of the mechanical potential means, where significant differences were found between insular populations and the other groups (see Table S3 in the Supplementary Materials).

Between-group mean comparisons identified significant differences in the linear measurements (PCL; CDR Y MRL), both angles and centroid size (*p* < 0.05 in all cases; see Table S3 in the Supplementary Materials). Regarding size, the smallest size was found in insular populations (maximum mandible length and centroid size), while the largest was in *T. e. elegans*. No significant differences were found for either measurements of the mandibular corpus width (ACW:PCW) (Table S3 in the Supplementary Materials).

The PERMANOVA (linear measurements) also showed significant differences between the three groups (see Supplementary Materials, Table S3). When the shape was used to compare the islands and populations, the discriminant analyses for the right hemimandible showed a classification rate of 92.9% when comparing island—*T. e. coquimbensis* (*p* = 0.0008) and 78.6% when comparing island—*T. e. elegans* (*p* < 0.0001). For the left hemimandibles, the rates were 61.5% for island—*T. e. coquimbensis* (*p* = 0.003) and 92.3% for island—*T. e. elegans* (*p* < 0.0001) (see Table S4 and Table S5, respectively, in the Supplementary Materials). In order to compare with the linear measurements, we found that, after creating the discriminant function, the rate was 77.08% (Jackknifed) for the left hemimandibles and 82.22% (Jackknifed) for the right hemimandibles, when comparing the three groups simultaneously (see Table S6 and Table S7, respectively, in the Supplementary Materials).

**Figure 6:**
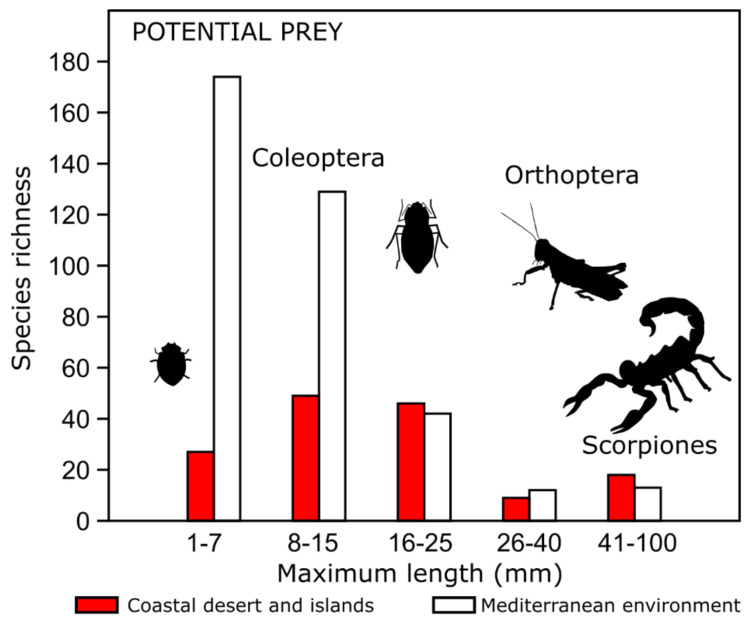
Arthropod prey richness by size. Coastal desert (*T. e. coquimbensis* and insular populations) and Mediterranean environment (*T. e. elegans*).

Arthropod prey abundance by size is shown in Figure 6, with small (8–15 mm) and medium (16–25 mm) species being predominant in the coastal desert environment, whereas, in the Central Mediterranean region, very small arthropods (1–7 mm) represent the largest proportion of species.

The comparison of fruit and seed abundance in both environments showed a lower proportion of fleshy fruits in the coastal desert (10%), which belonged to cactaceae of the genera *Copiapoa* and *Eulychinia*. In contrast, in the Mediterranean region, a larger proportion of fleshy fruits was found (31%), including drupes and berries from trees like *Cestrum parqui*, *Schinus molle* and *Azara dentata*, among others.

The correlations between vegetable cover index (NDVI) and rainfall (bio12) were positive with the centroid size per locality for both right and left hemimandibles in the vegetable cover indexes (NDVI) and rainfall (bio12) (see Table S3 in the Supplementary Materials). No significant correlation was found for the mean annual temperature (bio1) and centroid size.

## 4. Discussion

This article confirmed that the use of a geometric morphometrics tool was useful for the comparison of mandible shapes of the Chilean native Opossum *Thylamys elegans.*

It is important to notice that no differences were found in the mandible sizes between insular and continental populations at the same latitude (comparison of the insular population—*T.* *e. coquimbensis*) (see Table S3 in the Supplementary Materials) in this study, which opposes the classic “island rule”, proposing that the micromammal size increases in islands due to the absence of predators and stronger interspecific competition [7]. However, this general rule has been scarcely evaluated in Didelphidae [9]. In the islands used in this study, high predation by the burrowing owl (*Athene cunicularia*) on *Thylamys elegans* has been described [32], which may have influenced the mandible size patterns observed here.

Both island area and distance to the continent are regulatory factors in the island rule model (Lomolino 2005), since they would affect the resource availability and presence of predators. The RNPH archipelago is comprised of small islands (area < 600 Ha) located at a distance between 0.4 and 8 km from the continent [32,35], keeping the presence of predator species in both places (e.g., *Athene cunicularia*), which may explain our results regarding the absence of differentiation in size.

The results of the linear morphometrics analyses showed size differences between populations of the coastal desert and the central zone, consistent with the results of Palma [61] on the larger size of the subspecies *T. e. elegans.* The same results were also retrieved from the multivariate regression analysis, where a significant influence of the size was observed on the shape (see Figure S1 in the Supplementary Materials). Resources from islands may be less diverse and more limited than in continental environments [8,62]. This island diversity pattern may become more apparent in desertic environments, intensifying differentiations between populations, even between locations with similar environmental characteristics. The populations analyzed in this study showed clear differences in the mandibular shape and their biomechanical function (Figure 3 and Figure 5); this shape difference may be explained by a combination of selective forces that have been previously associated with types of diets [18,52], effects of body sizes [16,19] and genetic differences [17].

The mouse opossum feeds mainly on arthropods, with a lower proportion of seeds, fruits and small vertebrates [26,27,61,63]. Some studies have suggested a higher relevance of fruits, seeds and insect larvae in the composition of their diet [26,64], which may be associated with the environmental availability of such resources. In our study, these characteristics were present in all the individuals analyzed, with variations in the shapes and sizes of the insular populations, which have a more robust shape and a lower inclination of the coronoid process.

Another potential factor affecting the mandible shape might be a more arboreal behavior, which has been related to a higher curvature of the basicranium due to a higher encephalization process and reduced mouth opening. The latter aspect can be offset by an increased mandibular angle (angle A), allowing the mouth to open faster and wider [45,46]. This characteristic was observed in *T. e. elegans*, which had the largest angle A and whose distribution includes environments with more vegetation cover (shrubs and sclerophyll forests) and a higher presence of fleshy fruits [65,66] (Table 1). These characteristics suggest a potential arboreal behavior, which, in turn, might be reflected in the significant correlation between the mandible centroid size and environmental and bioclimatic variables, such as vegetation cover (NDVI) and mean annual rainfall (bio12).

The coastal desert populations (islands and continent) inhabit environments with poor vegetation cover and a low quantity of fleshy fruits [31,34,67]. Additionally, in this area, a higher availability of large prey (10–100 mm maximum length) has been reported, including tenebrionids, scorpions, lizards and geckos [32,67,68,69,70,71,72,73]. Regarding mandible shape, these populations present more robust mandibular morphologies, with deeper masseteric fossae, shorter molar lines, reduced retromolar fossae and a more perpendicular mandibular ramus (lower angle A), all in a smaller mandible. These mandible shapes could be related to a stronger bite force [37,46] and, thus, more efficient capturing and consumption of harder/tougher prey [37,38]. The mechanical potential is mainly associated with a stronger bite force, and surprisingly, it was lower in insular populations. However, this model only estimates the force exerted by the temporalis muscle [52].Since insular populations also present a deeper and more developed masseteric fossa (indicative of a larger masseter complex and, thus, of stronger bite forces from that muscle), a more developed masseter complex in these populations might offset the reduced bite force related to a lower MP [18].

The interpretation of the biomechanical model allowed us to suggest that mouse opossums from the islands have a smaller gape (related to a reduction of the temporalis muscle) but can close their mouths faster (inversely related with the MP) [74], which is effective to catch mobile and hard prey smaller in size. The differences found in the MP suggest that resources in islands change compared to the continent. However, further studies of the trophic resources available in these two areas of the coastal desert are required (islands vs. continent).

The morphological differences found between the insular and continental populations may be explained by differences in the prey consumption and/or availability, as observed in other insectivorous predators in this system (*Athene cunicularia)* that present a high consumption of vertebrates and functional responses associated with the differences in the availability of prey on islands [32]. Another relevant factor to be considered is the temporal separation between populations: the temporal separation between subspecies *T. e. elegans* and *T. e. coquimbensis* and other related lineages on the continent might be close to 1.84–1.28 Ma BP [24], but no major morphological differences between them were observed. In this context, however, the morphological differences found in the insular populations become more relevant, since these islands may have separated “recently” from the continent due to changes in the sea level (>100 m) that took place after the last glacial cycle (35 Kyr BP) [14,75].

## 5. Conclusions

Our results suggest that insularity influences morphology and feeding ecology, even though the populations analyzed (island and continent) share similar environments and prey. The main morphological and biomechanical differences were found in the insular populations. These differences might have been preserved due to the distance and geographic barriers that make the flow of individuals between the islands and the continent improbable. The specific characteristics observed in insular *Thylamys elegans* suggest an interesting differentiation process, which could require a reevaluation of the taxonomic status of these populations. These differences raise the question of whether the effect of insularity on morphology may be modulated by natural selection.

## Figures and Tables

**Table 1:** Summary of the seed and fruit richness in the coastal desert and Mediterranean region.

	Coastal Desert		Mediterranean Region	
Type of Fruit	No. Species	%	No. Species	%
Fleshy	4	10	29	31
Not Fleshy	36	90	67	69

## Data Availability

Not applicable.

## Supplementary Materials

The following supporting information can be downloaded at https://www.mdpi.com/article/10.3390/ani12091179/s1, Table S1. Summary of individuals used in each analysis, Table S2. Eingeenvalues, Principal componente analyses, Table S3. Results of T analyses, Table S4. Discriminant analysis with multivariate geometric morphometrics data. Left side., Table S5. Discriminant analysis with multivariate geometric mophometrics data. Right side, Table S6. Discriminant analyses with linear measurements. Left side, Table S7. Discriminant analyses with linear measurements. Right side, Figure S1. Multivariate regression, Figure S2. Ressidual matriz, Principal component analysis (PCA).
