# Supplementary material for: Insularity and Aridity as Drivers of Mandibular Disparity in Thylamys elegans (Waterhouse, 1839) from Populations of the Atacama Desert, Chile
Source: Animals (Basel). 2022 May 4;12(9):1179. doi: 10.3390/ani12091179 (PMC9100870; doi:10.3390/ani12091179)

SUPPORTING INFORMATION

Table S1. Summary of individuals used in each analysis. GM: Geometric Morphometrics MP: Mechanical potential; LM: Linear measurements

					GM		MP		LM		
**CODE**	**LOCALITY**	**LEFT**	**RIGHT**	**CATEGORY**	**LEFT**	**RIGHT**	**LEFT**	**RIGHT**	**LEFT**	**RIGHT**	COORDENATES
CHA	isla Chañaral	13	6	Island population	11	6	10	4	13	6	29°02'21.69''S;71°34'12.38''O
CHO	isla Choros	3	6	Island population	2	6	2	4	3	5	29°15'53.38'S; 71°32'24,10''O
IGV	isla Gaviota	1	3	Island population	0	2	1	2	1	3	29°15'40.04''S;71°28'48.71''O
PCC	Punta Choros	2	2	*T.e.coquimbensis*	2	2	2	2	2	2	29°14'49.09''S;71°27'28.62''O
PAC	Pan de Azúcar	0	1	*T.e.coquimbensis*	0	1	0	1	0	1	26°09'45.74''S;70°39'08.99''O
SMC	Salamanca	1	1	*T.e.coquimbensis*	1	1	1	1	1	1	31°47'2.35''S;70°57'10.30''O
VIL	Los Vilos	2	2	*T.e.coquimbensis*	1	1	1	1	2	1	31°54'43.87''S;71°30'40.33''O
QLP	Quebrada de la plata	15	15	*T. e.elegans*	14	15	14	14	15	13	33°29'40.80''S; 70°54'38.28''O
VLP	Valparaíso, Peñuelas	3	3	*T. e.elegans*	3	3	3	3	3	3	33°08'57.90''S; 71°33'37.31''O
MEL	El melón	5	4	*T. e.elegans*	4	4	5	4	5	4	32°42'08.94''S;71°13'11.85''O
BAT	Batuco	1	1	*T. e.elegans*	1	1	1	1	1	1	33°11'49.86''S; 70°50'8.77''O
PAN	Panul	2	3	*T. e.elegans*	2	3	2	3	2	3	33°32'06.21''S; 70°33'02.98''O
	TOTAL	48	47		41	45	42	40	48	43	

Table S2. Eingeenvalues, Principal componente analyses.

CA eigeevalues left side (Geometric morphometrics)				PCA eigeevalues right side (Geometric morphometrics)			
	Eigenvalues	% Variance	Cumulative %	Eigenvalues	% Variance	Cumulative %	
1.	0,00053574	30,408	30,408	1.	0,00055059	30,876	30,876
2.	0,00032199	18,276	48,684	2.	0,00033404	18,732	49,608
3.	0,00024678	14,007	62,69	3.	0,00022942	12,865	62,474
4.	0,0001519	8,622	71,312	4.	0,00015064	8,448	70,921
5.	0,0000926	5,256	76,568	5.	0,00011743	6,585	77,507
6.	0,00008045	4,566	81,134	6.	0,00008202	4,6	82,106
7.	0,00007098	4,029	85,163	7.	0,00005891	3,304	85,41
8.	0,00005538	3,143	88,306	8.	0,0000518	2,905	88,315
9.	0,00003493	1,982	90,289	9.	0,00003648	2,046	90,361
10.	0,00003185	1,808	92,097	10.	0,0000315	1,766	92,127
11.	0,00002788	1,583	93,679	11.	0,00002655	1,489	93,616
12.	0,0000201	1,141	94,82	12.	0,00002333	1,308	94,924
13.	0,00001897	1,076	95,896	13.	0,00001958	1,098	96,022
14.	0,00001817	1,031	96,928	14.	0,00001446	0,811	96,833
15.	0,00001365	0,775	97,703	15.	0,00001203	0,675	97,508
16.	0,00001043	0,592	98,295	16.	0,00001055	0,592	98,1
17.	0,00000658	0,373	98,668	17.	0,00000753	0,422	98,522
18.	0,00000613	0,348	99,016	18.	0,00000726	0,407	98,929
19.	0,00000446	0,253	99,269	19.	0,00000635	0,356	99,285
20.	0,00000399	0,226	99,495	20.	0,00000415	0,232	99,517
21.	0,00000294	0,167	99,662	21.	0,00000366	0,205	99,723
22.	0,00000221	0,126	99,788	22.	0,00000166	0,093	99,816
23.	0,0000019	0,108	99,895	23.	0,00000146	0,082	99,898
24.	0,00000099	0,056	99,951	24.	0,00000103	0,058	99,955
25.	0,00000063	0,036	99,987	25.	0,00000054	0,03	99,986
26.	0,00000023	0,013	100	26.	0,00000025	0,014	100

Table S3. Results of T analyses (right side). **i** = insular; **c** = coquimbensis; **e** = elegans

LEFT SIDE				RIGHT SIDE			
Comparation	Measure	Analyses	P	Comparation	Measure	Analyses	P
Island - T. elegans	Ángulo A	t-test	**0,03**	Islas - T. elegans	Ángulo A	t-test	**0,03**
Island - T. elegans	Ángulo B	t-test	**0,007**	Islas - T. elegans	Ángulo B	t-test	**0,001**
Island - T. elegans	MP	t-test	**0,03**	Islas - T. elegans	MP	t-test	**0,007**
Island - T. elegans	Centroide	t-test	**0,016**	Islas - T. elegans	Centroide	t-test	**0,0003**
Island - T. elegans	PCL	t-test	**0,002**	Islas - T. elegans	PCL	t-test	**0,0001**
Island - T. elegans	CDR	t-test	**0,0001**	Islas - T. elegans	CDR	t-test	**0,0001**
Island - T. elegans	MRL	t-test	**0,0003**	Islas - T. elegans	MRL	t-test	**0,003**
Island - T. elegans	ACW	t-test	0,1	Islas - T. elegans	ACW	t-test	0,3
Island - T. elegans	PCW	t-test	0.6	Islas - T. elegans	PCW	t-test	0,1
Island - T. elegans		PERMANOVA	**0,02**	Islas - T. elegans		PERMANOVA	**0,02**
LEFT SIDE				RIGHT SIDE			
Comparation	Measure	Analyses	P	Comparation	Measure	Analyses	P
Island - T.coquimbensis	Ángulo A	t-test	**0,00005**	Islas - T.coquimbensis	Ángulo A	t-test	**0,02**
Island - T.coquimbensis	Ángulo B	t-test	**0,03**	Islas - T.coquimbensis	Ángulo B	t-test	**0,008**
Island - T.coquimbensis	MP	t-test	**0,002**	Islas - T.coquimbensis	MP	t-test	**0,002**
Island - T.coquimbensis	Centroide	t-test	0,66	Islas - T.coquimbensis	Centroide	t-test	0,66
Island - T.coquimbensis	PCL	t-test	0,7	Islas - T.coquimbensis	PCL	t-test	0,9
Island - T.coquimbensis	CDR	t-test	**0,02**	Islas - T.coquimbensis	CDR	t-test	0,4

Island - T.coquimbensis	MRL	t-test	**0003**	Islas - T.coquimbensis	MRL	t-test	0,5
Island - T.coquimbensis	ACW	t-test	**0,01**	Islas - T.coquimbensis	ACW	t-test	0,2
Island - T.coquimbensis	PCW	t-test	0,6	Islas - T.coquimbensis	PCW	t-test	0,7
Island - T.coquimbensis		PERMANOVA	**0,002**	Islas - T.coquimbensis		PERMANOVA	**0,04**
LEFT SIDE				RIGHT SIDE			
Comparation	Measure	Analyses	P	Comparation	Measure	Analyses	P
T. e. elegans - T.coquimbensis	Ángulo A	t-test	**0,00008**	T. e. elegans - T.coquimbensis	Ángulo A	t-test	**0,003**
T. e. elegans - T.coquimbensis	Ángulo B	t-test	**0,00009**	T. e. elegans - T.coquimbensis	Ángulo B	t-test	**0,001**
T. e. elegans - T.coquimbensis	MP	t-test	0,22	T. e. elegans - T.coquimbensis	MP	t-test	0,42
T. e. elegans - T.coquimbensis	Centroide	t-test	**0,026**	T. e. elegans - T.coquimbensis	Centroide	t-test	**0,007**
T. e. elegans - T.coquimbensis	PCL	t-test	**0,003**	T. e. elegans - T.coquimbensis	PCL	t-test	**0,003**
T. e. elegans - T.coquimbensis	CDR	t-test	0,27	T. e. elegans - T.coquimbensis	CDR	t-test	**0,05**
T. e. elegans - T.coquimbensis	MRL	t-test	0,7	T. e. elegans - T.coquimbensis	MRL	t-test	**0,12**
T. e. elegans - T.coquimbensis	ACW	t-test	0,5	T. e. elegans - T.coquimbensis	ACW	t-test	0,2
T. e. elegans - T.coquimbensis	PCW	t-test	0,6	T. e. elegans - T.coquimbensis	PCW	t-test	0,3
T. e. elegans - T.coquimbensis		PERMANOVA	**0,002**	T. e. elegans - T.coquimbensis		PERMANOVA	**0,0003**
PEARON CORRELATION, LEFT SIDE				PEARSON CORRELATION, RIGHT SIDE			

Island - T.coquimbensis – T e.elegans	CS – Bio 12	Correlación	**0,04**	Islas - T.coquimbensis – T e.elegans	CS – Bio 12	Correlación	**0,007**
Island - T.coquimbensis – T e.elegans	CS – Bio 1	Correlación	0,15	Islas - T.coquimbensis – T e.elegans	CS – Bio 1	Correlación	**0,002**
Island - T.coquimbensis – T e.elegans	CS – NDVI	Correlación	**0,03**	Islas - T.coquimbensis – T e.elegans	CS – NDVI	Correlación	**0,004**

Table S4. Discriminant analysis with multivariate geometric morphometrics data. Left side.

p = 0,0031				
group	**island**	***coquimbensis***	Total	% Clasif.
**island**	8	5	13	61,5
coquimbensis	3	1	4	75

p = 0,0001				
group	**island**	***elegans***	Total	% Clasif.
**islas**	12	1	13	92,3
elegans	1	23	24	95,8

0,0625				
group	***coquimbensi***	***elegans***	Total	% Clasif.
coquimbensi	2	2	4	50
elegans	11	13	24	54,16

Table S5. Discriminant analysis with multivariate geometric mophometrics data. Right side

p = 0,0009				
group	**island**	***coquimbensis***	Total	% Clasif.
**island**	13	1	14	92,85
coquimbensis	1	4	5	80

p = 0,0001				
group	**island**	***elegans***	Total	% Clasif.
**island**	11	3	14	78,57
elegans	1	25	26	96,15

0,1365				
group	***coquimbensi***	***elegans***	Total	% Clasif.
coquimbensi	2	3	5	40
elegans	6	20	24	83,33

Table S6. Discriminant analyses with linear measurements. Left side

% correctly classificated, Jackknifed 77,08				
	**island**	***coquimbensis***	***elegans***	Total
**island**	14	2	1	17
Coquimbensis	0	3	2	5
elegans	2	4	20	26
**Total**	16	9	23	48

Table S7. Discriminant analyses with linear measurements. Right side

% correctly classificated, Jackknifed, 82,22				
	**island**	***coquimbensis***	***elegans***	Total
**island**	10	4	0	14
coquimbensis	2	2	1	5
elegans	1	0	25	26
**Total**	13	6	26	45

Figure S1.a. Multivariate regressión left side

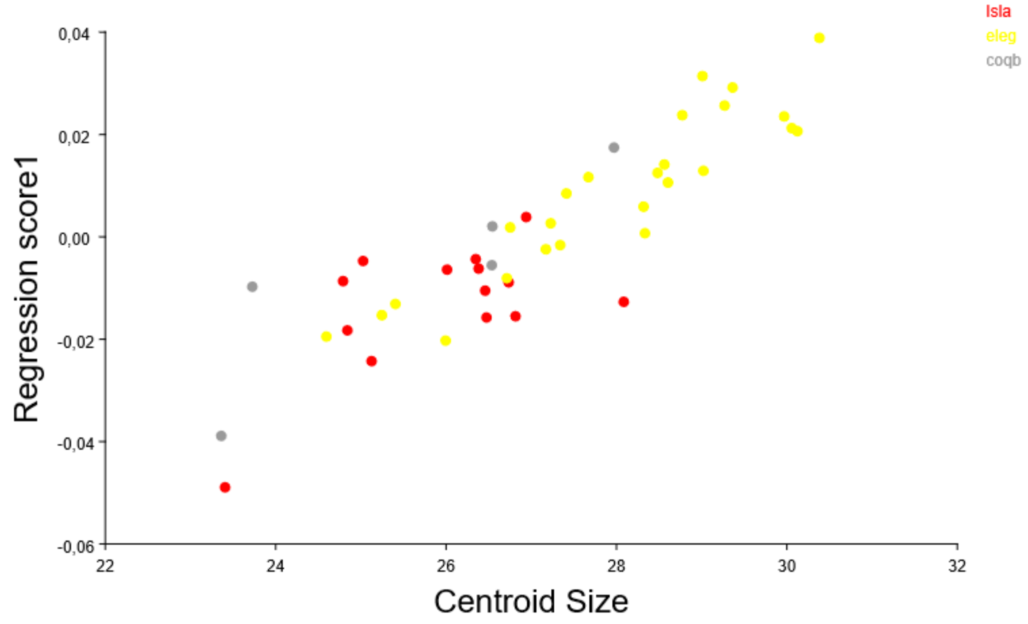

Figure S1. b. Multivariate regressión right side

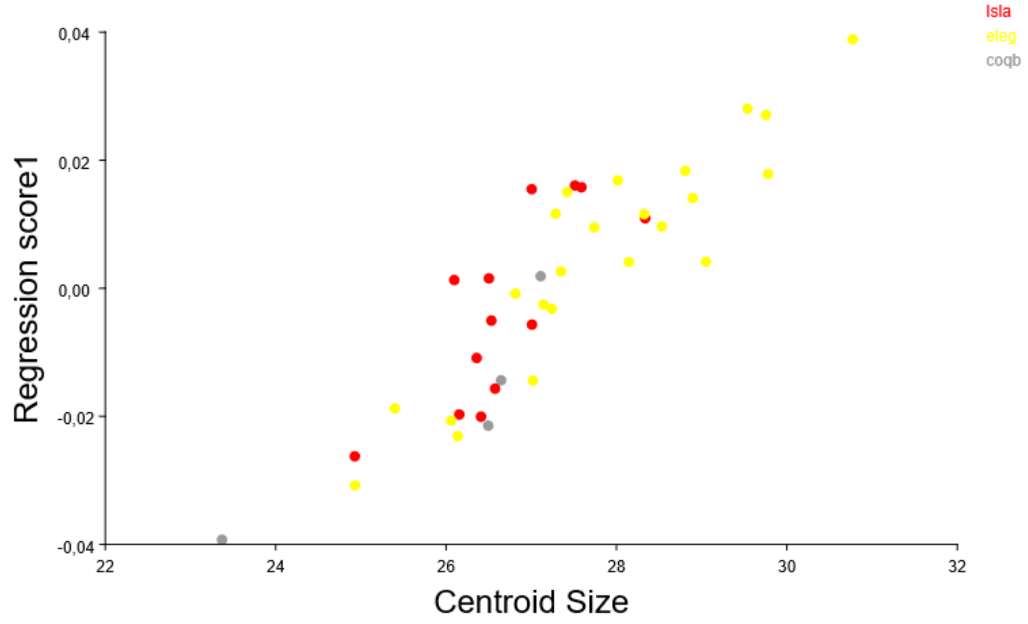

Figure S2.a Ressidual matriz, *Principal component analysis (PCA)* left side

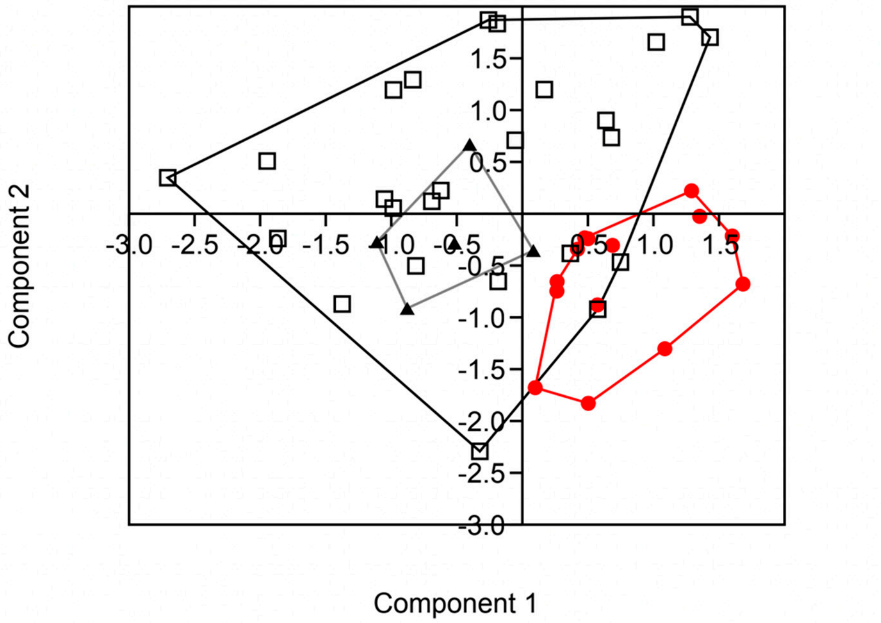

Figure S2.b Ressidual matriz, *Principal component analysis (PCA)* right side

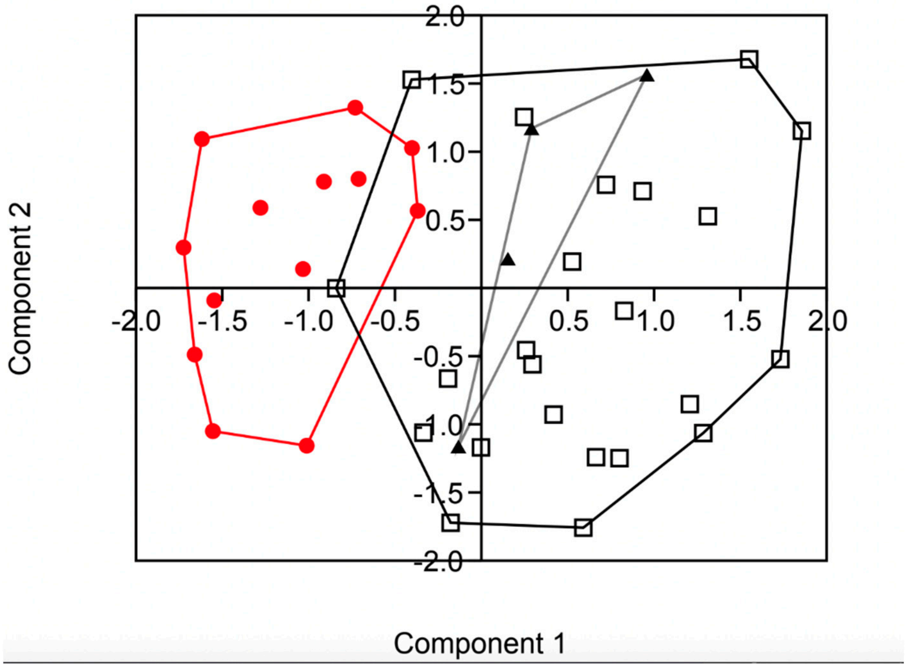

Supplement: Supplementary file 1 [file animals-12-01179-s001.zip › animals-1675098-supplementary.pdf]
